# ESRRG and PERM1 Govern Mitochondrial Conversion in Brite/Beige Adipocyte Formation

**DOI:** 10.3389/fendo.2020.00387

**Published:** 2020-06-12

**Authors:** Sebastian Müller, Aliki Perdikari, Dianne H. Dapito, Wenfei Sun, Bernd Wollscheid, Miroslav Balaz, Christian Wolfrum

**Affiliations:** ^1^Institute of Food, Nutrition and Health, Department of Health Sciences and Technology (D-HEST), ETH Zürich, Zurich, Switzerland; ^2^Institute of Translational Medicine, Department of Health Sciences and Technology (D-HEST), ETH Zürich, Zurich, Switzerland; ^3^Life Science Zurich Graduate School, Molecular Life Sciences Program, Zurich, Switzerland

**Keywords:** beige adipocytes, brown adipocyte, adipogenesis, estrogen receptor, adipose tissue

## Abstract

When exposed to cold temperatures, mice increase their thermogenic capacity by an expansion of brown adipose tissue mass and the formation of brite/beige adipocytes in white adipose tissue depots. However, the process of the transcriptional changes underlying the conversion of a phenotypic white to brite/beige adipocytes is only poorly understood. By analyzing transcriptome profiles of inguinal adipocytes during cold exposure and in mouse models with a different propensity to form brite/beige adipocytes, we identified ESRRG and PERM1 as modulators of this process. The production of heat by mitochondrial uncoupled respiration is a key feature of brite/beige compared to white adipocytes and we show here that both candidates are involved in PGC1α transcriptional network to positively regulate mitochondrial capacity. Moreover, we show that an increased expression of ESRRG or PERM1 supports the formation of brown or brite/beige adipocytes *in vitro* and *in vivo*. These results reveal that ESRRG and PERM1 are early induced in and important regulators of brite/beige adipocyte formation.

## Introduction

White and brown adipose tissue have divergent functions. White adipocytes store chemical energy within a single lipid droplet to release it back to the body when needed. In contrast, brown adipocytes primarily convert chemical energy into heat through the action of uncoupling protein 1 (UCP1) (1, 2). It is known that brown adipocytes do not only reside in a distinct fat pad in the interscapular region in rodents, but are also found interspersed between white adipocytes in other anatomical locations (3, 4). The latter have been termed brite (“brown-in-white”) or beige adipocytes and are identified by their multilocular appearance and the expression of UCP1 (5). Their formation can be induced through a plethora of stimuli, most prominently through exposure of the animals to a cold environment, by beta-adrenergic stimulation (6) and other endocrine factors (7, 8). Experimental evidence suggests that the arising brite/beige adipocytes can derive from *de novo* differentiation of stem cells (9, 10) as well as by direct interconversion of mature adipocytes (11–13).

The orphan nuclear estrogen related receptor gamma (ESRRG) is highly expressed in energy dependent tissues such as brain, heart, skeletal muscle, kidney and BAT and is responsible for cell-type specific function in the majority of those tissues (14). Studies have shown that ESRRG is not important for brown preadipocyte differentiation but for brown adipocyte function, acting in a complementary way together with other ESRR members in mitochondrial biogenesis and oxidative function (15). In fact, it was shown that ESRR members mediate the adaptive response of BAT to adrenergic stimulation (16). Moreover, ESRRG is necessary for the induction of UCP1 in *in vitro* differentiating cells (17) and for the maintenance of BAT thermogenic activity *in vivo* (18). ESRRG was shown to interact with GADD45γ to regulate UCP1 levels during cold adaption of brown adipose tissue (19). In regards to browning of white adipose tissue, the 1-benzyl-4-phenyl-1H-1,2,3-triazole derivative is shown to enhance the function of ESRRG and increase browning *in vitro* and *in vivo* (20). The potential transcription factor PGC1 and ESRR-induced regulator in muscle 1 (PERM1) has not been described in the context of adipose tissue so far, but was recently reported to regulate OXPHOS proteins in muscle *in vitro* and *in vivo* (21, 22). As ESRRG, PERM1 acts downstream of PGC1α, a master-regulator of mitochondrial capacity (23).

We aimed to study the latter process and showed that as early as 24 h after a cold stimulus, several known browning markers are significantly upregulated in the white adipocyte fraction. Importantly, we identified ESRRG and PERM1 as novel transcriptional regulators of brite/beige adipocyte formation.

## Results

### Upregulation of Esrrg and Perm1 Expression in the Inguinal White Adipose Tissue Mature Adipocyte Fraction of 129/SV Mice Upon 24 h of Cold Exposure

**Figure 1:**
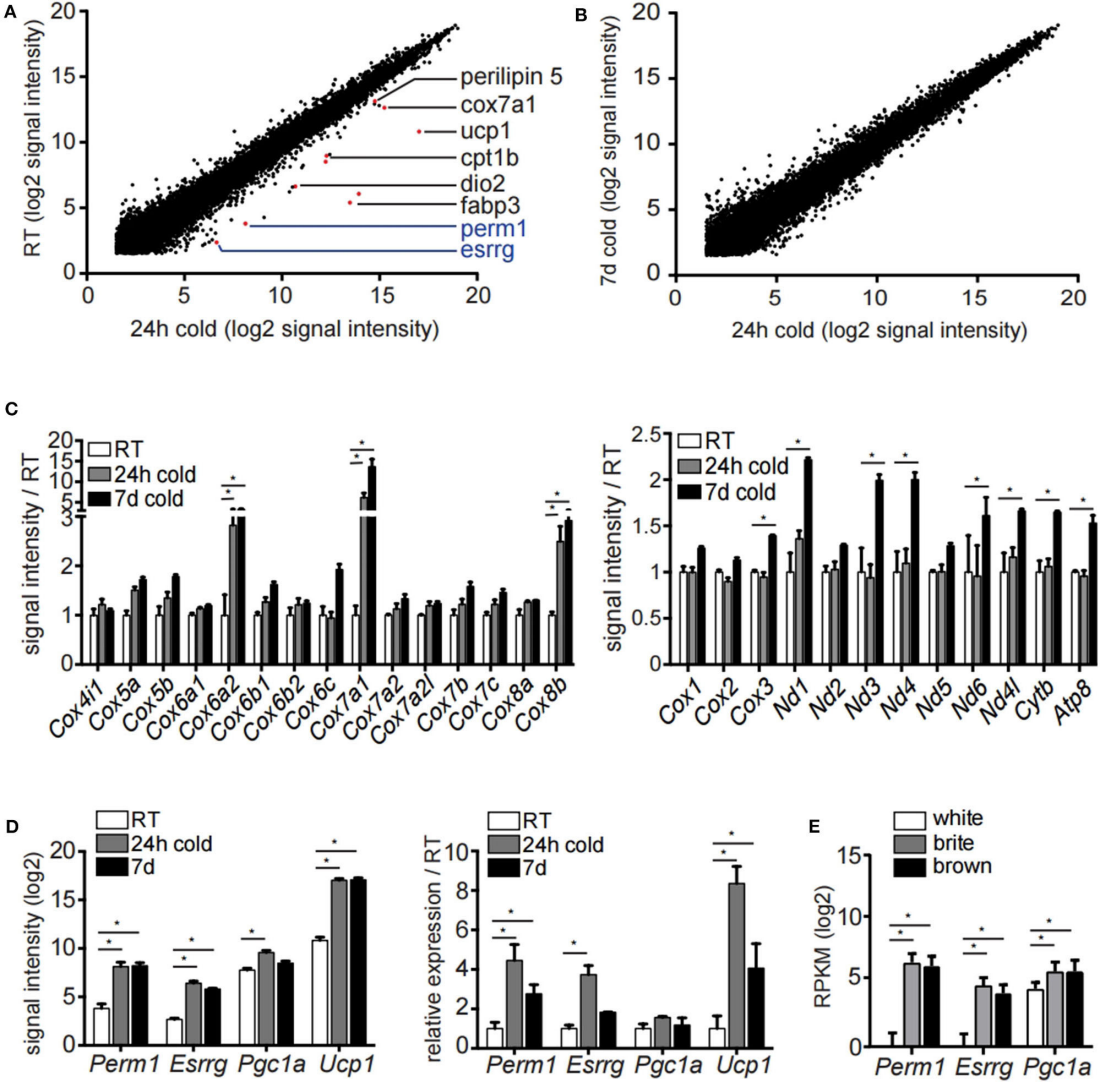
Microarray analysis of inguinal white adipocytes during cold exposure. **(A,B)** Signal intensities (log2) of all probes measured of the extracted RNA of the adipocyte fraction of ingWA comparing 24 h cold exposure and room temperature **(A)** or 24 h and 7 days cold exposure **(B)**. Depicted in red are browning markers upregulated after cold treatment. **(C)** Signal intensity of specific nuclear encoded mitochondrial genes for OXPHOS complex IV (left) and all mitochondrial encoded genes (right) in room temperature, after 24 h and 7 days of cold exposure. Data are presented as microarray signal intensities relative to room temperature as mean with SEM (*n* = 3–5). **(D)** Expression of target genes *Perm1, Esrrg, Pgc1*α, and *Ucp1* in room temperature, after 24 h and 7 days of cold exposure shown in the microarray (left) and in an independent cohort of mice via qRT-PCR (right). Data are presented as signal intensities (log2) or relative expression (ddCt) compared to room temperature, respectively, as mean with SEM (*n* = 3-5 per cohort). **(E)** Expression of target genes *Perm1, Esrrg*, and *Pgc1*α in FACS sorted mature adipocytes according to GFP expression under control of the *Ucp1* promoter. Data are presented as mean RPKM (log2) with SD (*n* = 10 per cell type). Statistical testing (*t*-test) was performed between room temperature condition and cold housing for 24 h or 7 days, respectively, or between sorted white adipocytes and brite or brown adipocytes. A statistically significant result, meaning *p*-value < 0.05, is indicated with an asterisk (^*^).

To study the transcriptional events underlying the appearance of brite/beige adipocytes we housed 129/SV mice at 8°C for 24 h, 7 days or kept them at room temperature. In order to identify transcriptional changes induced by acute and prolonged cold exposure, we performed transcriptomic analysis of the mature inguinal white adipocyte (ingWA) fraction using RNA microarray. We excluded from further analysis two out of five individual mice housed at room temperature which already expressed high levels of *Ucp1* (Figure S1), a phenomenon previously described in literature (24). Intriguingly, on the transcriptome level, the browning program was fully activated already after 24 h of cold exposure, as demonstrated by the upregulation of common browning makers *Dio2, Fabp3, Cpt1b, Perilipin 5, Cox7a1*, and *Ucp1* (Figure 1A). Even though there is a clear difference in the multilocular adipocyte content (Figure S1B), the transcriptomic changes between 24 h and 7 days of cold exposure were only marginal (Figure 1B), indicating that the transcriptional program for brite/beige adipocyte formation is kept constant after the initial activation. A key feature in the appearance of brite/beige adipocytes is the increase in mitochondrial capacity (25, 26). We found specific nuclear encoded mitochondrial genes upregulated already after 24 h of cold exposure, indicating mitochondrial remodeling. On the other hand, mitochondrial encoded genes were only regulated after 7 days of cold exposure, which likely reflects the bulk increase in mitochondrial mass (Figure 1C). Analysis of early transcriptional changes revealed two genes, *Esrrg* and *Perm1*, to be significantly upregulated after 24 h of cold exposure (Figure 1A). We confirmed the expression of *Esrrg* and *Perm1* by qRT-PCR in ingWA fraction 24 h after cold exposure in an independent cohort of mice (Figure 1D). We further validated the *Esrrg* and *Perm1* expression in pure populations of mature adipocyte isolated by fluorescent activated cell sorting (FACS, Figure S1C). We used transgenic mice in the C57BL/6 background expressing green-fluorescent protein (GFP) under the control of the *Ucp1* promoter housed at 8°C for 7 days. RNA expression analysis revealed *Perm1* exclusive expression in the brite/beige adipocyte fraction, while *Esrrg* expression was enriched in the brite/beige compared to the white adipocytes (Figure 1E).

### Transcriptome Analysis Shows That Esrrg and Perm1 Expression Positively Correlates With Browning Markers

**Figure 2:**
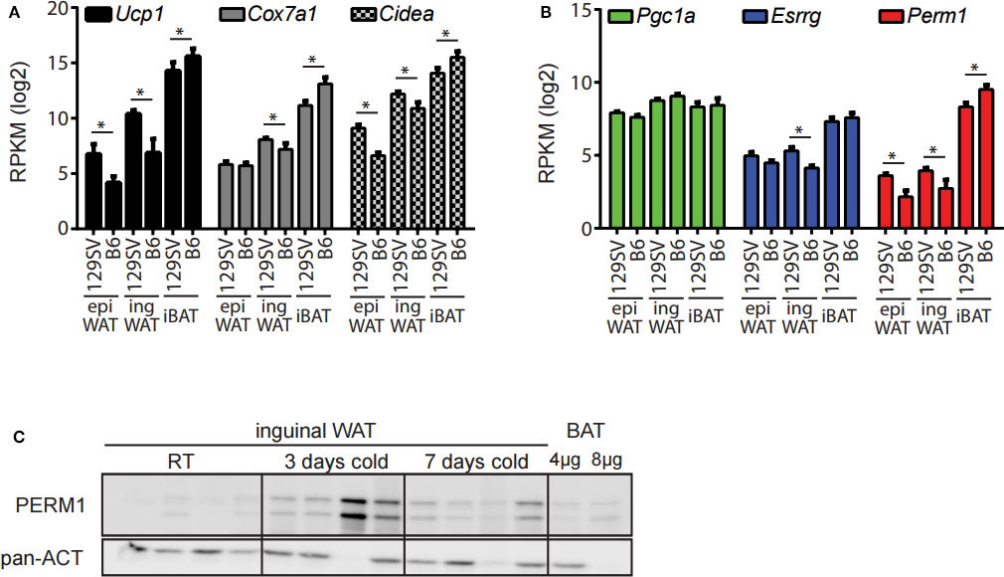
*Esrrg* and *Perm1* mirror thermogenic capacity in adipocyte fractions of 129/SV and C57BL/6 mouse models. **(A)** Expression of common browning markers *Ucp1, Cox7a1*, and *Cidea* in the adipocyte fractions of epiWA, ingWA, and iBA in 129/SV or C57BL/6 mice housed at room temperature. Data are presented as mean RPKM with SEM (*n* = 4). **(B)** Expression of *Pgc1*α*, Esrrg, and Perm1* in the adipocyte fractions of epiWA, ingWA and iBA in 129/SV or C57BL/6 mice housed at room temperature. Data are presented as mean RPKM with SEM (*n* = 4). **(C)** PERM1 and UCP1 protein levels in ingWAT of 129/SV mice at room temperature and after 3 and 7 days of cold exposure. BAT at 1/10 and 1/5 of protein loaded shown for comparison. Each lane represents a single mouse (*n* = 4). Statistical testing (*t*-test) was performed between the mouse strains (129SV & B6) in each of the different tissue derived adipocyte fractions. A statistically significant result, meaning *p*-value < 0.05, is indicated with an asterisk (^*^).

The 129/SV mouse strain has a higher propensity of browning in the ingWA compared to C57BL/6 (27, 28). Therefore, we analyzed if this phenotypic difference can be recapitulated through whole transcriptome analysis. The adipocyte fractions of the ingWA, epididymal white (epiWA) and interscapular brown adipose tissue (iBA) were isolated from both strains housed at room temperature and analyzed by next-generation RNA sequencing. Classical browning markers *Ucp1, Cox7a1*, and *Cidea* were nearly absent in epiWA of C57BL/6 with very low expression in the 129/SV mouse model (Figure 2A), while they were expressed higher in the ingWA of 129/SV mice, indicating the presence of few brite/beige cells already at room temperature. Interestingly, we detected browning markers at higher levels in the iBA of C57BL/6 in comparison to 129/SV, potentially indicating a compensatory mechanism, to keep the total brown/brite/beige capacity constant. The expression levels of *Esrrg* and *Perm1* accurately correlated with browning markers in the two mouse models (Figure 2B), suggesting a possible role in brite/beige and brown adipocytes. To validate that changes on the transcript level also result in increased protein abundance, we cold-exposed 129/SV mice for 3 and 7 days, respectively. We could show that PERM1 is not detectable at room temperature but is expressed after cold stimulation in ingWA (Figure 2C). Due to the unavailability of ESRRG specific antibodies, we could not confirm protein expression of ESRRG.

### Perm1 mRNA and Protein Is Expressed in High Energy Demanding Tissues

**Figure 3:**
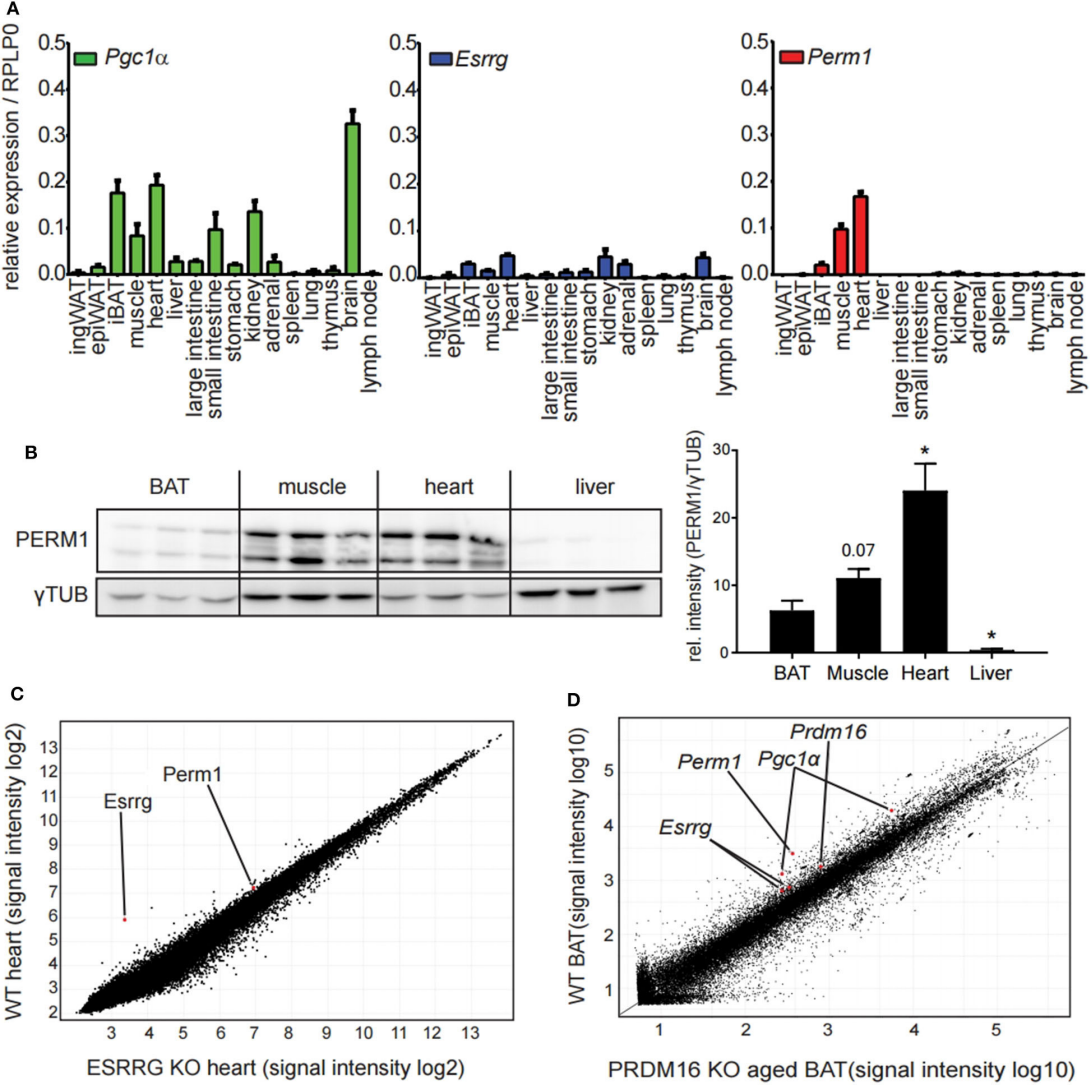
PERM1 expression is confined to tissues with a high energy demand and with ESRRG involved in energy metabolism. **(A)** Expression of *Perm1, Esrrg*, and *Pgc1*α in a panel of 16 tissues derived from C57BL/6 mice housed at room temperature. Data are shown as relative expression to reference gene RPLP0 with SEM (*n* = 5). **(B)** Representative western blot and quantification of PERM1 in BAT, muscle, heart, and liver. Each lane represents a single mouse (*n* = 3). **(C)** Microarray analysis of RNA extracted from heart tissue of ESRRG knockout mice as described in (29). Data were retrieved from GSE8199, plotted are all transcripts as log2 signal intensities (*n* = 3). **(D)** Representative microarray analysis of RNA extracted from BAT of aged mice, where PRDM16 is knocked out under the control of MYF5 promoter, leading to a whitening of BAT, as described in (31). Data were retrieved from GSE55080, depicted is one representative microarray with all probes as signal intensities (total *n* = 4). A statistically significant result, meaning *p*-value < 0.05, is indicated with an asterisk (^*^).

To characterize further the candidate genes *Esrrg* and *Perm1*, we analyzed the expression pattern in a tissue panel of C57BL/6 mice housed at room temperature. Interestingly, while we detected broad expression of *Pgc1*α and *Esrrg, Perm1* expression was confined to tissues with a high energy demand, in particular iBAT, skeletal muscle and heart (Figure 3A), confirmed also on protein level (Figure 3B). The importance of ESRRG and PERM1 in energy metabolism is evident in global ESRRG knockout mice, which die shortly after birth, due to a heart-defect, as the organ cannot sustain its function despite an increase in mitochondrial number (29). In this study, *Perm1* is considerably downregulated, in heart tissue of the *Esrrg* knockout mice (Figure 3C). Moreover, a recent study by Harms and colleagues describes a whitening of brown adipose tissue upon knockout of *Prdm16*, a master regulator of the brown adipocyte program (30), in mature brown adipocytes (31). Consistently, in the publicly available dataset, *Perm1* is also among the highest downregulated proteins in the whitened BAT of these mice (Figure 3D).

### Esrrg and Perm1 Show Low Expression in *in vitro* Differentiated Adipocytes and Abundance Was Increased Upon Adenoviral Overexpression of Pgc1a

**Figure 4:**
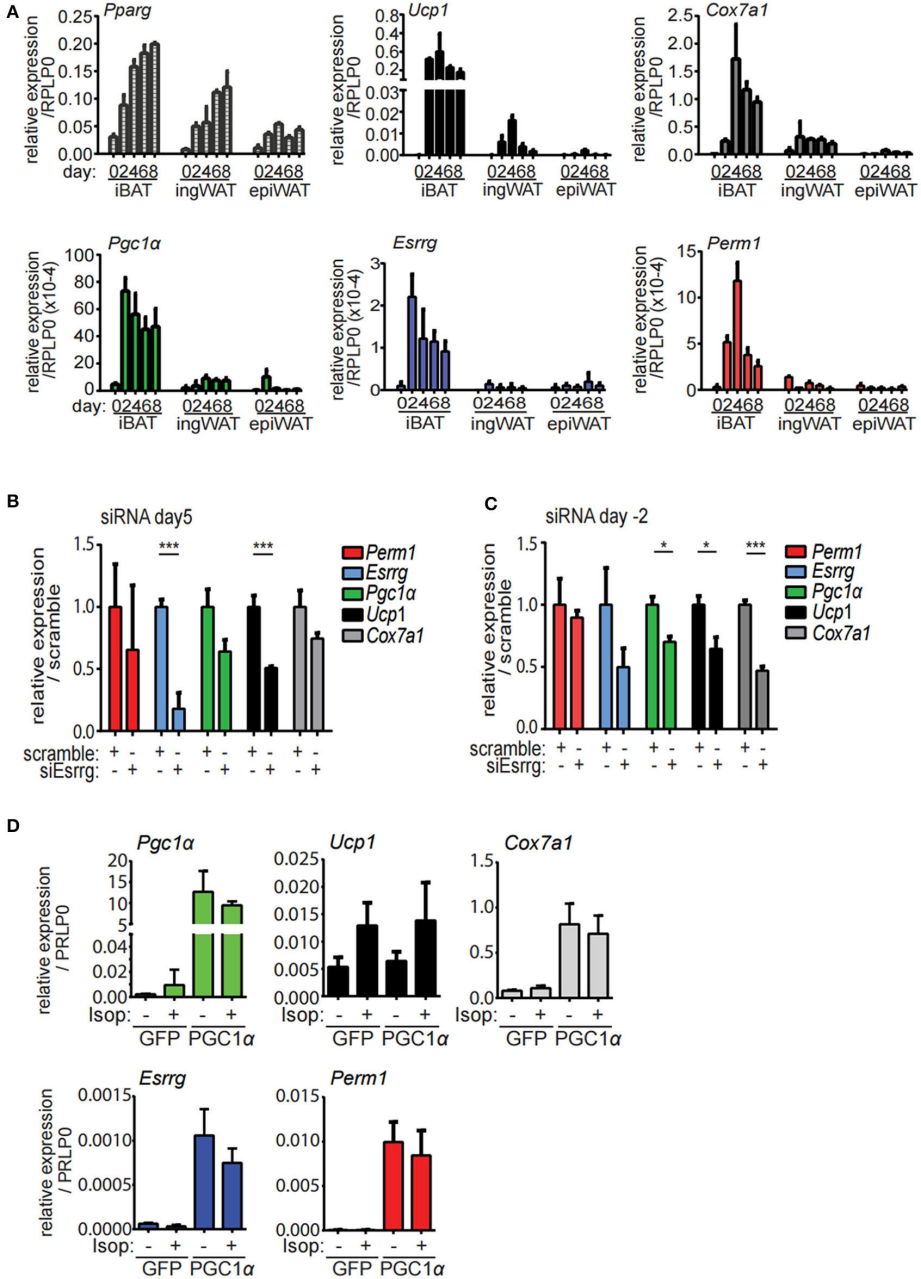
*Esrrg* and *Perm1* have low expression levels *in vitro* but can be induced in a PGC1α dependent manner. **(A)** Time course of *in vitro* brite/beige adipocyte differentiation of SVF derived from iBA, ingWA or epiWA of 129/SV mice. *Pparg* (all isoforms) is shown as general adipocyte marker, *Ucp1*, and *Cox7a1* as markers for brown adipocytes. Data are presented as relative expression to reference gene RPLP0 with SEM (*n* = 3). **(B)** Gene expression in immortalized brown mature adipocytes *in vitro* differentiated and treated with siRNAs targeting *Esrrg* at day 5 of differentiation. Data are presented as relative expression (ddCt) relative to non-targeting siRNA as mean with SD (*n* = 3). **(C)** Gene expression in immortalized brown pre-adipocytes treated with siRNAs targeting *Esrrg 2* days prior to induction of differentiation. RNA was extracted on day 8 after induction and data are presented as expression (ddCt) relative to non-targeting siRNA as mean with SD (*n* = 3). **(D)** Gene expression in immortalized brown mature adipocytes *in vitro* differentiated and infected with adenovirus to overexpress PGC1α or GFP at day 5 after induction. Data are presented as relative expression to reference gene RPLP0 with SD (*n* = 3). A statistically significant result, meaning *p*-value < 0.05 and < 0.001, is indicated with an asterisk (^*^) and (^***^), respectively.

In order to establish an *in vitro* model to characterize the function and the role of PERM1 and ESRRG in brite/beige adipocyte formation, we isolated stromal-vascular fraction (SVF) of the epiWA, ingWA and iBA depots of 129/SV mice and subjected them to *in vitro* brite/beige adipocyte differentiation (10). To assess the dynamics of *Perm1* and *Esrrg* expression during the differentiation of SVF cells into adipocytes, we harvested cells every 2 days starting from induction (day 0). *Ppar*γ, as a general adipocyte marker, indicated adipocyte differentiation of cells derived from all three tissues, while browning markers *Ucp1* and *Cox7a1* were only detected in ingWA-derived SVF cells and were strongly induced in iBA-derived SVF cells during the differentiation time course (Figure 4A). While *Pgc1*α expression levels resembled these observations, *Esrrg* and *Perm1* could not be detected in ingWA-derived differentiating cells and were induced at very low levels in the iBA derived differentiating SVF cells, indicating that these cells are not a suitable model to study PERM1 and ESRRG function. In conclusion, while *Esrrg* and *Perm1* are strong indicators of browning capacity *in vivo*, their expression *in vitro* is limited.

To functionally characterize our candidate proteins, we used immortalized brown pre-adipocytes, which can be differentiated to mature adipocytes *in vitro* (32). *Esrrg* and *Perm1* were silenced in proliferating preadipocytes (day−2) or in mature adipocytes (day 5), to study their role in regulation of brown/beige adipocyte formation and thermogenic activity, respectively. SiRNA mediated knockdown of *Esrrg* in mature brown adipocytes at day 5 of differentiation led to a significant reduction in *Ucp1* and *Cox7a1*, while *Pgc1*α was not affected (Figure 4B), indicating that ESRRG drives brown adipocyte thermogenic activity. Moreover, siRNA mediated repression of *Esrrg* in preadipocytes prior to the induction of differentiation (siRNA treatment at day−2) resulted in significantly reduced expression of *Ucp1, Cox7a1*, and *Pgc1*α as well as *Esrrg* in mature adipocytes, indicating a role of *Esrrg* during the early phase of adipogenic differentiation (Figure 4C). Altogether, these data indicate that ESRRG controls both formation as well as the thermogenic activity of brown/beige adipocytes. We were unable to achieve knockdown of *Perm1* with siRNA treatment; hence, no change in expression of other genes was observed (Figure S2A). As the expression levels of *Esrrg* and *Perm1 in vitro* are low, we aimed to increase their levels and analyze the concurrent functional implications. We employed an adenoviral system to overexpress either PGC1α or green fluorescent protein (GFP) as a control [kindly provided by Prof. Christoph Handschin (33)], as PGC1α was shown to increase *Perm1* and *Esrrg* abundance in muscle studies (21). We demonstrated that *Perm1* and *Esrrg* could be upregulated in a *Pgc1*α dependent manner in *in vitro* differentiated mature brown adipocytes (Figure 4D). Interestingly, *Cox7a1* was co-regulated, while *Ucp1* levels were unaffected. The latter can be explained by *Ucp1* expression being already affected by control adenovirus treatment compared to untreated cells (Figure S2C). Moreover, the combination of adenoviral and siRNA treatment resulted in non-differentiating cells (data not shown). Notably, adenoviral overexpression of PGC1α in immortalized pre-adipocytes already lead to the expression of known target genes *Esrra* and *Cox7a1* in a *Pgc1*α-dependent manner, but not *Esrrg* or *Perm1* (Figure S2D).

### Expression of Perm1 in a Doxycycline Induced Stable Cell Line Leads to Increased OXPHOS Protein Expression

**Figure 5:**
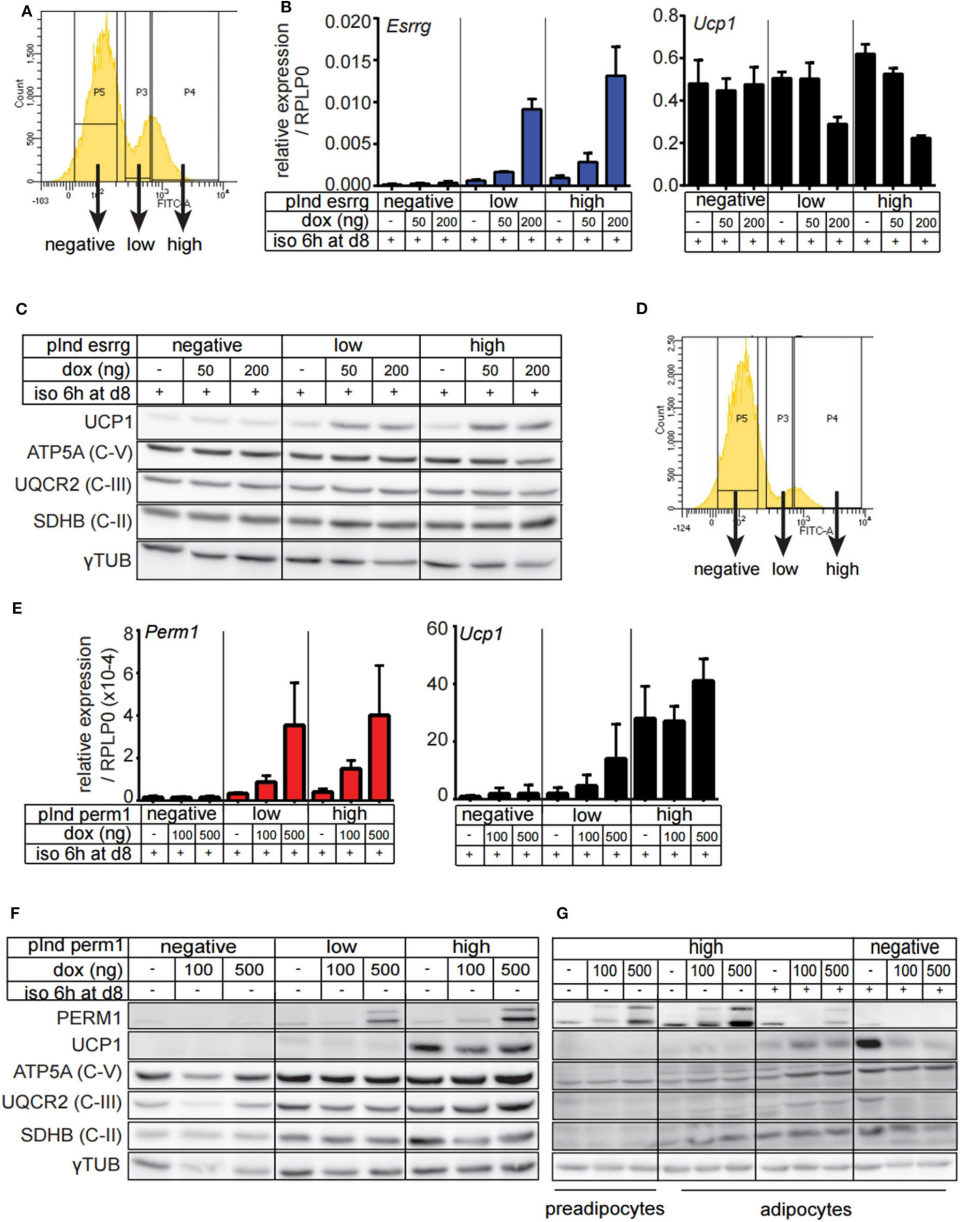
Generation and characterization of brown adipocyte cell lines expressing either ESRRG or PERM1 in a doxycycline dependent manner. **(A)** Representative FACS plots from immortalized brown pre-adipocytes transduced with lentiviral particles containing pInd21_ESRRG to polyclonally generate new cell lines where ESRRG is not expressed or is lowly or highly induced by doxycycline treatment, respectively. **(B)** *Esrrg* and *Ucp1* expression in *in vitro* differentiated pInducer_ESRRG cell lines, treated with doxycycline from day 5 to 8. Data are presented as relative expression to reference gene *RPLP0* with SD (*n* = 3). **(C)** UCP1, ATP5A, UQCR2, and SDHB expression in *in vitro* differentiated pInducer_ESRRG cell lines, treated with doxycycline from day 5 to 8. Gray dotted line indicates removal of the marker lane from the digital blot image. **(D)** Representative FACS plots from immortalized brown pre-adipocytes transduced with lentiviral particles containing pInd21_PERM2 to polyclonally generate new cell lines where PERM1 is not expressed or is lowly or highly induced by doxycycline treatment, respectively. **(E)** *Perm1* and *Ucp1* expression in *in vitro* differentiated pInducer_PERM1 cell lines treated with doxycycline from day 5 to 8. Data are presented as relative expression to reference gene *RPLP0* with SD (*n* = 3). **(F)** UCP1, ATP5A, UQCR2, and SDHB protein levels in *in vitro* differentiated pInducer_PERM1 cell lines, treated with doxycycline from day 5 to 8. Gray dotted line indicates removal of the marker lane from the digital blot image. **(G)** UCP1, ATP5A, UQCR2, and SDHB expression in pInducer_PERM1 cell lines treated in preadipocyte state with doxycycline for 96 h or *in vitro* differentiated and treated with doxycycline from day 4 to 8. Gray dotted lines indicate removal of the marker lane from the digital blot image.

Since endogenous expression of *Esrrg* and *Perm1* is low *in vitro*, we set out to establish a model system, which does not rely on adenoviral transduction and allows for targeted expression of either *Esrrg* or *Perm1*. Therefore, we used the lentiviral pInducer21 system (34) to express *Esrrg* or *Perm1* under the control of doxycycline, including GFP as a positive selection marker for transduced cells. Immortalized brown preadipocytes were infected and FACS sorted according to their GFP signals, to polyclonally generate six cell lines having negative, low or high GFP expression, corresponding to their ability to express either ESRRG or PERM1 upon doxycycline treatment. As a next step, we differentiated the pInducer_Esrrg cell lines (Figure 5A) in the presence of doxycycline during the last 3 days of differentiation. Indeed, we could show the effective induction of *Esrrg*, which inversely correlated with *Ucp1* mRNA expression (Figure 5B). On the protein level, UCP1 was increased in an *Esrrg*-dependent manner, while the representative proteins of the oxidative phosphorylation chain (OXPHOS) were not affected (Figure 5C). Similar experiments with the pInducer_Perm1 cell lines demonstrated an efficient induction of PERM1 on transcript and protein level, while UCP1 remained unaffected (Figures 5D,E). Interestingly, components of the OXPHOS showed a trend to be positively affected by PERM1 expression. To corroborate this finding, we repeated the experiment with a prolonged doxycycline treatment (last 4 days of differentiation) of the pInducer_Perm1 cell lines. The omission of doxycycline during the 6-h isoproterenol stimulation led to a significant decrease in induced *Perm1* mRNA (Figure S3A) and protein levels. Moreover we observed the recently described adverse effects of doxycycline on mitochondrial stability, by inhibiting the mitochondrial ribosome (35). Despite these confounding effects, we demonstrated an increase in representative OXPHOS proteins in a PERM1 dependent manner (Figures 5F,G). Moreover, these experiments did not reveal a direct regulation of *Perm1* by *Esrrg* or vice versa (Figure S3B).

### *In vivo* Overexpression of PERM1 Correlates With Increase in Levels of Selective OXPHOS Components

**Figure 6:**
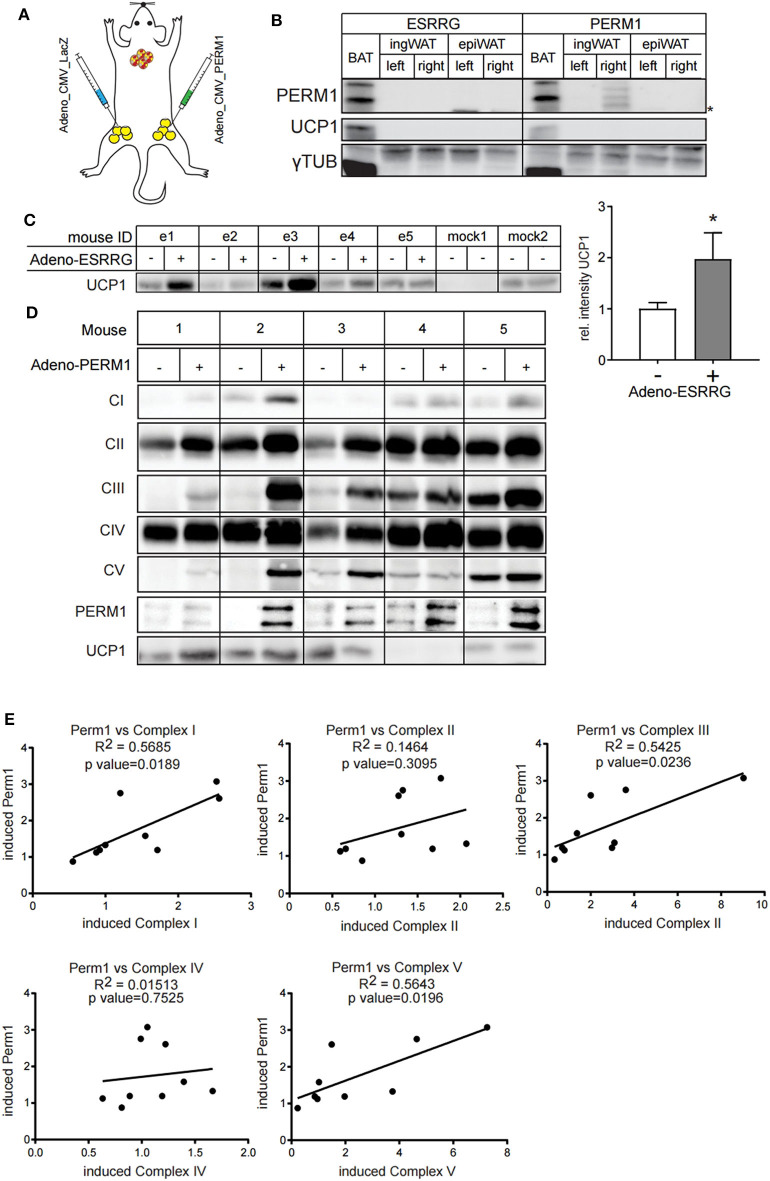
ESRRG and PERM1 positively regulate browning of ingWAT *in vivo*. **(A)** Schematic illustration of the direct injection of adenoviral particles leading to the overexpression of LacZ (left ingWA fat pad) and PERM1 (right ingWA fat pad) in the same mouse. **(B)** PERM1 detection in the ingWA fat pad injected with the adenoviral particles for PERM1 overexpression. **(C)** Representative western blot and quantification of UCP1 in the ingWA fat pad injected with adenoviral particles for ESRRG overexpression (+). **(D)** Representative blots for OXPHOS complex subunits, UCP1 and PERM1 expression in ingWA fat pad injected with the adenoviral particles of PERM1 overexpression. **(E)** Correlation of PERM1 expression with different OXPHOS subunits as shown by protein expression in nine mice injected in one ingWA fat pad with adenoviral particles for PERM1 overexpression. A statistically significant result, meaning *p*-value < 0.05, is indicated with an asterisk (^*^).

Since ESRRG and PERM1 can positively regulate brown adipocyte function *in vitro*, we aimed to investigate whether their overexpression also supports browning of white adipose tissue *in vivo*. Therefore, we directly injected adenoviruses into the right ingWA fat-pad of 129/SV mice, which led to the overexpression of either *Esrrg* or *Perm1*, and control virus for LacZ expression into the left ingWA fat pad of the same mouse (Figure 6A). Two days after injection, our target proteins were only expressed locally, and we could not detect a spread to liver on transcript (Figure S4) and protein level (Figure 6B). Two days after the injections, we exposed the animals to 8°C for 7 days to induce browning of ingWA. Intriguingly, despite varying degrees of browning in the individual animals, we observed an asymmetric browning between the fat pads injected with virus overexpressing *Esrrg* and the control virus, supporting that *Esrrg* overexpression lead to increased levels of UCP1 *in vivo* (Figure 6C). *Perm1* overexpression on the other hand, led to a selective increase in Complex 1, 3, and 5 of the OXPHOS, while UCP1 levels remained rather constant (Figures 6D,E). In summary, based on our data we propose that ESRRG and PERM1 support browning *in vitro* and *in vivo*. While ESRRG regulates UCP1 abundance, PERM1 controls the amount of the OXPHOS components.

## Discussion

During cold exposure, mice must increase their thermogenic capacity to maintain their body temperature by increasing brown adipose tissue mass and forming brite/beige adipocytes within white adipose tissue depots (36, 37). Experimental evidence demonstrates that brite/beige adipocytes can arise from *de novo* differentiated stem cells (9, 10) or by conversion of mature adipocytes in the ingWA (12, 13). In this study, we solely analyzed the floating adipocyte fraction after cold exposure, thereby excluding cold induced transcriptional changes in stromal cells, as is described in whole tissue analyses (38). We could show that common browning factors are already fully upregulated on a transcriptional level after 24 h of cold stimulation, while morphological and UCP1 protein expressing brite/beige adipocytes are only detectable after several days of cold exposure, depending on the mouse model used (9, 12, 25). Mitochondria are the main functional organelles for non-shivering thermogenesis (26). Based on our transcriptomic profile, we detected a selective upregulation of specific OXPHOS components after 24 h of cold exposure, indicating remodeling concurrent with expansion of mitochondria, during the conversion of a phenotypic white to a phenotypic brite/beige cell. Similar observations have been made in ultra-structural analyses of mitochondria during cold adaption in inguinal white adipocytes (39). On a proteomic level, it has been shown, that brown fat mitochondria rather resemble those of muscle than of white adipose tissue (40). Intriguingly, ESRRG and PERM1, the candidates we propose to govern this phenotypic conversion, are important regulators of mitochondrial capacity in muscle tissues (22, 41).

The orphan nuclear receptor ESRRG has already been shown to be necessary for the induction of UCP1 in *in vitro* differentiating cells (17) as well as for maintaining BAT thermogenic activity *in vivo* (16, 18). We corroborated these results by showing that knockdown of *Esrrg* in brown preadipocytes or in mature brown adipocyte *in vitro* leads to a decrease in UCP1 expression. This data is consistent with the phenotype of adipocyte-specific *Esrrg* knockout mice, which display marked downregulation of BAT-selective genes, pronounced whitening of BAT and cold intolerance (18). Moreover, it has recently been shown that ESRRG interacts with GADD45γ to regulate UCP1 levels during cold adaption of brown adipose tissue (19). According to our data, ESRRG exerts a similar function in regulating UCP1 induction during the formation of brite/beige adipocytes. Cold exposure of 129/SV mice for 24 h showed increased levels of *Esrrg* and *Ucp1* in the mature adipocyte fraction of the ingWAT.

The potential transcription factor PERM1 has not been described in the context of adipose tissue so far, but was recently reported to regulate OXPHOS proteins in muscle *in vitro* and *in vivo* (21, 22). We observed very low expression levels of PERM1 in brown adipocytes *in vitro* similar to a study comparing *in vitro* differentiated C2C12 cells to primary murine muscle, where PERM1 was more than 100-fold less abundant in C2C12 cells (42).

ESRRG and PERM1 act downstream of PGC1α, a master-regulator of mitochondrial capacity (23). Since PGC1α is highly regulated post-transcriptionally (43, 44), the transcript level alone is of limited informational value. When we analyzed the transcriptional profiles of the adipocyte fractions of different adipose tissue depots in 129/SV and C57BL/6 mice, we found that although *Pgc1a* expression is similar between the two strains and different depots, *Esrrg* and *Perm1* expression accurately mirrors the increased thermogenic capacity of 129/SV mice. Moreover, we gathered further evidence that total thermogenic capacity is kept rather constant (45, 46) by demonstrating that C57BL/6 mice have higher expression of classical browning marker genes in interscapular brown adipocytes, while in 129/SV mice, expression of browning markers is distributed between interscapular brown and inguinal white adipocyte fractions. *In vivo*, we could show that overexpression of PERM1, although does not increase UCP1 expression, correlates with OXPHOS components regulation, suggesting an important role of PERM1 in mitochondrial function. Multiple reports indicate that regulating brite adipocyte formation is sufficient to elicit a metabolic phenotype (24, 47–49). Therefore, molecules inducing brown/beige adipocyte formation and activity such as irisin, BMP7, FGF21, natriuretic peptides and mineralocorticoid receptor antagonists have attracted a lot of attention in recent years (44, 50–54). Here, we show that ESRRG and PERM1 govern the phenotypic conversion of white to brite/beige adipocytes. However, future studies are needed to validate the therapeutic potential of these two transcriptional regulators in animal models of obesity.

By analyzing the transcriptome of adipocytes of different mouse models and inguinal adipocytes during cold exposure, we identified ESRRG and PERM1 as regulators of brite/beige adipocyte formation. Both ESRRG and PERM1 are under the control of the PGC1α transcriptional network and upon cold exposure, their expression is increased in ingWA. While ESRRG controls UCP1 levels, PERM1 is regulating the components of the oxidative phosphorylation chain and both processes together are key events in the phenotypic conversion of a white to a brite/beige adipocyte.

## Limitations of Study

Our paper lacks the exact molecular mechanism through which PERM1 and ESRRG modulate the browning process. We show descriptive evidence from several angles, but in the scope of this study lack the appropriate *in vivo* models, to show a mechanistic link to certain physiological functions. Future studies are needed to validate the metabolic impact of PERM1 and ESRRG on mitochondrial function and brown/beige adipocyte thermogenic activity.

## Experimental Procedures

### Chemicals and Reagents

All chemicals and reagents were obtained from Sigma-Aldrich, unless specified otherwise.

### Cloning and Virus Production

#### Cloning

Coding transcript sequences for *Esrrg* and *Perm1* were retrieved from Ensembl and synthesized (GenScript) in sequence verified puc57 carrier plasmids flanked by NruI and XhoI restriction sites (Perm1: 2455 residues; Esrrg: 1408 residues). Both constructs were cloned into pENTR1A_dual_selection (Invitrogen) entry vectors for further use.

#### Adenovirus Production

Adenoviral particles carrying overexpression constructs under control of the CMV promotor to express either GFP or PGC1α were a kind gift by Prof. Handschin and described elsewhere (55). Plasmids to produce adenoviral particles to overexpress ESRRG or PERM1 were generated with the pAd/CMV/V5-DEST Gateway Vector Kit (Invitrogen) by gateway cloning through a one-step LR-clonase (Invitrogen) reaction. Directing of insertion and nucleotide sequence of the insert was verified by sequencing analysis (Microsynth). Viruses were produced and passaged in HEK 293A cells (Invitrogen), purified with the Adenovirus Standard Purification ViraKit (Virapur) according to manufacturer‘s instructions and titrated by FACS analysis (GFP, PGC1α) or plague assays (ESRRG, PERM1, LacZ).

#### Lentivirus Production

The destination vector pINDUCER21 (ORF-EG) for lentivirus production was a gift from Stephen Elledge & Thomas Westbrook (Addgene plasmid # 46948) (34). ESRRG and PERM1 sequences were inserted into the pINDUCER21 plasmid by gateway cloning through a one-step LR-clonase (Invitrogen) reaction. Directing of insertion and nucleotide sequence of the insert was verified by sequencing analysis (Microsynth). Lentiviral particles were produced in HEK 293T cells (Thermo Fischer) by co-transfection of psPAX2 (a gift from Didier Trono; Addgene plasmid # 12260), pMD2.g (a gift from Didier Trono; Addgene plasmid # 12259) and pINDUCER21 plasmid in a 9:1:10 ratio using Lipofectamine 2000 (Thermo Fisher). Cells were incubated with DMEM supplemented with 10 % FBS, 1.1 % BSA and 5 mM sodium butyrate for 36 h. Supernatant was harvested, centrifuged for 5 min at 200 g to pellet cellular debris and filtered (0.45 μm). Before freezing the crude virus preparation, 5 μg/ml polybrene was added.

### Experimental Models

#### Mouse Work

129S2/SvPasCrl wild-type mice for all experiments were acquired from Charles-River at 4 weeks of age. C57BL/6N wild-type mice were bred in-house. *Ucp1-GFP* mice were previously described (12). All experiments were performed with young adult (12 weeks old) male mice kept on an inverted 12 h dark/light cycle and fed chow diet *ad libitum*. Cold stimulation was performed in temperature and humidity-controlled climate chambers (Memmert) with 4-5 animals housed in type II cages at an air-temperature of 8° C. All animal procedures were approved by the Veterinary office of the Canton of Zürich.

#### Virus Injection

Purified adenoviral particles were pre-incubated for 2 h at room temperature with 1.2 % poly-lysine. Animals were anesthetized by intra-peritoneal injection of 50 μl of a 1:1 mixture of Xylosine (1:5 in PBS) and Ketamine (1:2.5 in PBS). Adenoviruses (50 μl, 5.0 x 10^9^ PFU/ml per fat pad) were directly injected into the ingWAT fat pad through the skin.

#### Tissue Sampling

For tissue sampling, animals were euthanized in carbon dioxide atmosphere. Popliteal lymph nodes were carefully removed, and adipose tissue depots were dissected and snap frozen in liquid nitrogen. For tissue sampling after adenoviral injections, euthanized animals were perfused utilizing a peristaltic pump with PBS containing 5 mM EDTA.

#### Adipocyte and SVF Isolation

Dissected adipose tissues were minced and incubated in 5 ml collagenase buffer (25 mM KHCO_3_, 12 mM KH_2_PO_4_, 1.2 mM MgSO_4_, 4.8 mM KCl, 120 mM NaCl, 1.2 mM CaCl_2_, 5 mM glucose, 2.5% BSA, 2 mg/ml collagenase type II (clostridium histolyticum); sterile filtered (0.2 μm) for 60 min at 37 °C on a shaker and repeatedly re-suspended. The reaction was stopped by addition of 5 ml DMEM (Gibco) supplemented with 10 % FBS (Gibco) and 1 % Pen/Strep (Gibco) and centrifugation for 5 min at 200 g. The floating adipocyte fraction was carefully removed and filtered through 100 μm cell strainer (BD bioscience). Before further processing, the adipocyte fraction was washed three times by careful resuspension in separate tubes with 1 ml PBS (Gibco) followed by centrifugation for 2 min at 200 g. The SVF pellet was filtered through a 40 μm cell strainer (BD bioscience) and incubated in 1 ml erythrocyte lysis buffer (154 mM NH_4_lC, 10 mM KHCO_3_ and 0.1 m M EDTA) for 5 min at 24°C. After centrifugation for 5 min at 200 g, the SVF cells were re-suspended in DMEM supplemented with 10 % FBS and 1 % Pen/Strep.

#### Cell Sorting of Adipocyte Populations

FACS sorting of adipocytes according to their GFP expression was described previously (56). Briefly, cell sorting of adipocytes was performed using an Aria III high-speed sorter (BD Bioscience). A nozzle of 130 mm diameter, sheath pressure of 10 psi and a standard 4-way purity mask as described in the sorter manual was used during all sorts. Transgenic *Ucp1-eGFP* mice, expressing the eGFP protein under the control of *Ucp1* promoter, were cold acclimated at 8°C for 7 days and the mature adipocyte fractions from iBAT and iWAT were separated by FACS using eGFP. The adipocyte population was first defined in the forward and side scatter by size and internal complexity characteristics. The GFP^+^ population was defined in the respective gate and the mature brown adipocytes were isolated from the mature fraction of iBAT. The same strategy was applied for the mature adipocyte fraction of iWAT to isolate the GFP^+^ population, which constitutes brite adipocytes, and the adjacent GFP^−^ population that constitutes white adipocytes. For each sample, 3 or 6 same gender mice were pooled per sample and 500–3,000 cells were collected directly in RNA Lysis Buffer (Qiagen) and kept on ice until frozen and stored at −80°C.

#### Cell Culture Work

All cells were routinely passaged in complete DMEM (Gibco) supplemented with 10 % FBS (Gibco) and 1 % Pen/Strep (Gibco), unless stated otherwise. Isolated SVF cells were plated confluent at a density of 100 000 per well in a 96-well plate (day−2). Brown adipogenesis was induced (day 0) by addition of 5 mM dexamethasone, 0.5 mg/ml insulin, 0.5 mM isobutyl methylxanthine, 1 μM rosiglitazone and 1 nM T3 in complete DMEM. Two days after induction the medium was switched to complete DMEM supplemented with 0.5 mg/ml insulin and 1 nM T3, the medium was refreshed every other day until the cells were harvested (day 8). Immortalized brown pre-adipocytes as well as derived pINDUCER21_ESRRG and pINDUCER21_PERM1 cell lines were grown to confluence. Differentiation was induced the next day by addition of complete DMEM supplemented with 20 nM insulin, 0.5 mM isobutyl methylxanthine, 125 μM indomethacin, 1 μM dexamethasone, 1 nM T3 and 1 μM rosiglitazone. Two days after induction the medium was switched to complete DMEM supplemented with 20 nM insulin and 1 nM T3, consecutively the medium was refreshed every other day until the cells were harvested (day 8). Isoproterenol (1 μM) and doxycycline treatments of the pINDUCER cell lines were performed in addition, at the indicated time-points. To knockdown candidate proteins, 15 nmol siRNA smart-pools (4 siRNAs per gene, Dharmacon) were reverse-transfected into pre-adipocytes (day−2) or mature adipocytes (day 5) using Lipofectamine RNAiMAX (Invitrogen).

#### Adenoviral Infection

Adenoviral infections were performed at day 5 of differentiation. Purified viral particles were pre-incubated in complete DMEM supplemented with 1.2 % poly-lysine (vol/vol) for 2 h at room temperature, before being added to the culture at MOI of 10, if not indicated otherwise.

#### Lentiviral Infection and Cell Line Generation

For lentiviral infection of immortalized brown pre-adipocytes, crude virus preparation was added to sub-confluent cells and the culture plate centrifuged at 200 g for 10 min. After 1-h incubation at 37°C the crude virus was diluted 1:1 with complete DMEM. After 24 h the medium was changed to complete DMEM. The infected cells were passaged for 2 weeks and the integration of the pINDUCER construct verified by visible GFP expression through microscopy. Polyclonal FACS sorting was performed at the ETHZ Flow Cytometry Core Facility on a FACS Aria III (BD Biosciences) flow cytometer.

### Sample Preparation and Molecular Analyses

#### RNA Extraction

RNA extraction of adipocyte fractions from single mice for microarray analysis was performed using the RNeasy Mini Kit (Qiagen). For NGS analysis, adipocytes fractions of three mice were pooled and lysed in Trizol reagent (Life Technologies). Samples were centrifuged at 14,000 x g for 15 min and the interphase transferred to a new tube. After addition of chloroform, RNA in the colorless upper phase was purified with the RNeasy Mini Kit (Qiagen). From whole tissues and *in vitro* differentiated adipocytes, RNA was extracted with the Trizol reagent according to the manufacturer‘s instructions followed by DNase digestion (Invitrogen) and purification through NaOAc precipitation. RNA quantity and purity were controlled with a Nanodrop-2000 spectrophotometer. Of *in vitro* differentiated SVF cells and pInducer cell lines RNA extraction, DNase digestion and direct on-column cDNA synthesis was performed using the MultiMACS system (Miltenyi Biotec) according to the manufacturer's instructions.

#### cDNA Synthesis and Quantitative Real-Time PCR

Reverse transcription was performed using the High Capacity cDNA Reverse transcription kit (Applied Biosystems) with 1 μg RNA per reaction or the MultiMACS system (Miltenyi Biotec). QRT-PCR was performed with Fast Sybr Green Master Mix (Life Technologies) in 10 μl reactions in 96-well format on StepOnePlus (Applied Biosystems) or in 384-well on ViiA7 (Applied Biosystems) systems. All primers used are listed in Supplemental Table 1.

#### Microarray Analysis

RNA probe labeling, purification, hybridization to the microarray and scanning was performed by the Functional Genomics Center Zurich (FGCZ) utilizing the Mouse Gene Expression v2 4 × 44K, G4846A Microarray Kit (Agilent). Raw data extraction was performed by the FGCZ, in brief, the raw probe intensities were extracted with Agilent's Feature Extraction software (v9.5.3.1). Log2-transformation and normalization using the quantile normalization method were performed with R (v3.1). To find genes with significant expression changes between groups, empirical Bayes statistics were applied to the data by moderating the standard errors of the estimated values using the limma-package (v3.18) (57). *P*-values were obtained from the moderated t-statistic and corrected for multiple testing with the Benjamini–Hochberg method.

#### Next-Generation Sequencing Analysis

RNA sequencing was performed utilizing the HiSeq 2000 (Illumina) platform as previously described (56). Raw data was analyzed at the FGCZ. In brief, sequences were aligned to the Mus musculus reference genome (build GRCm38) and quantification of gene level expression was carried out using RSEM (Version 1.2.12) (58, 59). RNAseq data of pure murine white, brite/beige and brown adipocytes was previously published (56) and is available in the European Nucleotide Archive under accession number PRJEB20634.

#### Protein Extraction and Western Blotting

*In vitro* differentiated adipocytes were scraped off the culture plates in RIPA buffer [50 mM Tris pH 7.4, 150 mM NaCl, 5 mM EDTA, 1 % NP40, protease inhibitor cocktail (Roche)]. Muscle and heart tissues were powdered on dry ice using a mortar and pestle prior to re-suspension in RIPA. Lysates were further processed with a TissueLyser LT (Qiagen; 5 min at 50 Hz) and sonicated in an ice-cooled branson-type sonication bath for 30 min before being cleared of debris by centrifugation at 14,000 g for 15 min at 4°C. For adipose tissues, RIPA buffer was supplemented with 0.5 % sodium deoxycholate, 1 % Triton-X and 10 % glycerol and additionally centrifuged at 14,000 g for 15 min at 23°C to remove the liquid fat layer prior to clearing of debris. Protein concentration of the supernatants was determined either by DC Protein Assay (Bio-Rad) or Pierce BCA assay (Thermo Scientific). Equal amounts of proteins were separated on SDS-polyacrylamide gels in a Mini-Protean tetra (Bio-Rad) apparatus, before transfer to a nitrocellulose membrane (PerkinElmer). Antibodies used for probing were UCP1 (1:1000, ab10983, Abcam), PERM1 (C1orf170, 1:1000, HPA031711, Atlas Antibodies), total OXPHOS (1:500, ab110413, Abcam), pan-Actin (D18C11, 1:1000, CS#8456, Cell Signaling) and γ-tubulin (1:6000, T6557, Sigma-Aldrich). Chemiluminescent signals of the HRP-conjugated secondary antibodies (Calbiochem) were visualized by a LAS 4000 mini ImageQuant system (GE Healthcare Life Sciences).

### Quantification and Statistical Analysis

For *in vivo* studies, littermates were randomly assigned to the groups. Sample sizes were determined on the basis of previous experiments using similar methodologies. The animal numbers used for all experiments are indicated in the corresponding figure legends. All cell culture experiments were performed with 2–3 technical replicates for RNA and protein analysis, and independently reproduced 2–4 times. Results are reported as mean ± SEM or SD, as indicated in the figure legends. Two-tailed unpaired Student's *T*-test was applied on comparison of two groups. In case of non-normal data distribution, a non-parametric Wilcoxon test was performed. ANOVA was applied on comparisons of multiple groups. Pearson's correlation coefficient was calculated, and all statistical analyses were performed using GraphPad Prism 6 and R version 3.4.4. Statistical differences are indicated as ^*^for *P* < 0.05, ^**^for *P* < 0.01 and ^***^for *P* < 0.001.

## Data Availability Statement

All datasets generated for this study are included in the article/Supplementary Material.

## Ethics Statement

The animal study was reviewed and approved by Kantonales Vetamt Zürich.

## Author Contributions

SM and CW designed the study. BW and CW supervised the experiments. SM, AP, DD, and WS performed the experiments. SM and BW performed the proteome analysis. SM, AP, WS, and MB performed the transcriptome analysis. SM, MB, and CW wrote the paper. All authors reviewed and edited the manuscript. All authors contributed to the article and approved the submitted version.

## Conflict of Interest

The authors declare that the research was conducted in the absence of any commercial or financial relationships that could be construed as a potential conflict of interest.

## Supplementary Material

Figure S1 *Ucp1* expression in ingWA of individual mice during cold exposure. **(A)** *Ucp1* expression levels in the adipocyte fraction of ingWA of individual mice house at room temperature or cold exposed for 24 h or 7 days, respectively. Dotted line separates groups at room temperature with low or high *Ucp1* expression. Data is presented as log2 microarray signal intensity. **(B)** Representative immunohistochemical images of inguinal WAT (H&E stained) from a different cohort of mice housed at room temperature (RT), 24 h or 7 days at 8°C. Major morphological changes are visible after 7 days of cold exposure, only minor changes after 24 h. Micrographs were taken at 10x magnification. **(C)** Representative FACS plots from interscapular BAT (iBAT) and inguinal WAT (iWAT), and gating strategy for GFP^+^ brown adipocytes, GFP^+^ brite and GFP^−^ white adipocytes.

Figure S2 *In vitro* characterization of ESRRG and PERM1. **(A)** Gene expression in immortalized brown mature adipocytes *in vitro* differentiated and treated with siRNAs targeting *Perm1* at day 5 of differentiation. Data are presented as relative expression (ddCt) relative to non-targeting siRNA as mean with SD (*n* = 3). **(B)** Gene expression in immortalized brown mature adipocytes *in vitro* differentiated and treated with siRNAs targeting *Perm1 2* days prior to differentiation. Data are presented as relative expression (ddCt) relative to non-targeting siRNA as mean with SD (*n* = 3). **(C)** *Ucp1* expression in immortalized brown mature adipocytes *in vitro* differentiated and infected either with mock or with adenovirus to overexpress PGC1α or GFP at day 5 after induction. Data are presented as relative expression to reference gene RPLP0 with SD (*n* = 3). **(D)** Gene expression in immortalized brown mature adipocytes infected with different concentrations of adenoviral particles for PGC1α overexpression. Data are presented as relative expression to the lowest virus concentration (1/32 dilution; *n* = 1 per condition).

Figure S3 *Esrrg* and *Perm1* do not induce each other in *in vitro* differentiated brown adipocytes **(A)** *Perm1* expression after doxycycline is omitted during 6-h isoproterenol stimulation. Data are presented as relative expression to reference gene *RPLP0* as mean with SD (*n* = 3). **(B)** *Perm1* and *Esrrg* expression in in *in vitro* differentiated pInducer_ESRRG and pInducer_PERM1 cell lines, respectively, treated with doxycycline from day 5 to 8. Data are presented as relative expression to reference gene *RPLP0* as mean with SD (*n* = 3).

Figure S4 Overexpression of PERM1 or ESRRG by direct adenoviral injection is confined to the fat pad injected. **(A)** *Esrrg* or *Perm1* expression in the fat pad injected with the respective adenovirus. Data are presented as relative expression to reference gene *RPLP0* (*n* = 1 mouse per virus).
