# Supplementary figures and images for: ESRRG and PERM1 Govern Mitochondrial Conversion in Brite/Beige Adipocyte Formation
Source: Front Endocrinol (Lausanne). 2020 Jun 12;11:387. doi: 10.3389/fendo.2020.00387 (PMC7304443; doi:10.3389/fendo.2020.00387)

Figure S1

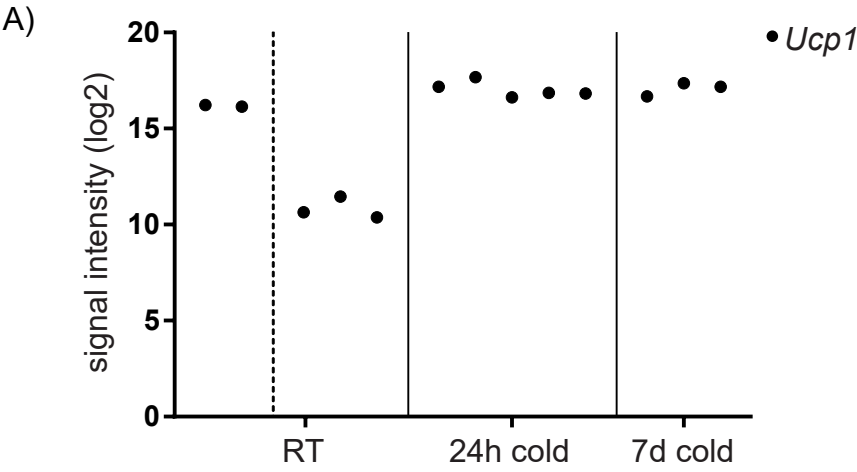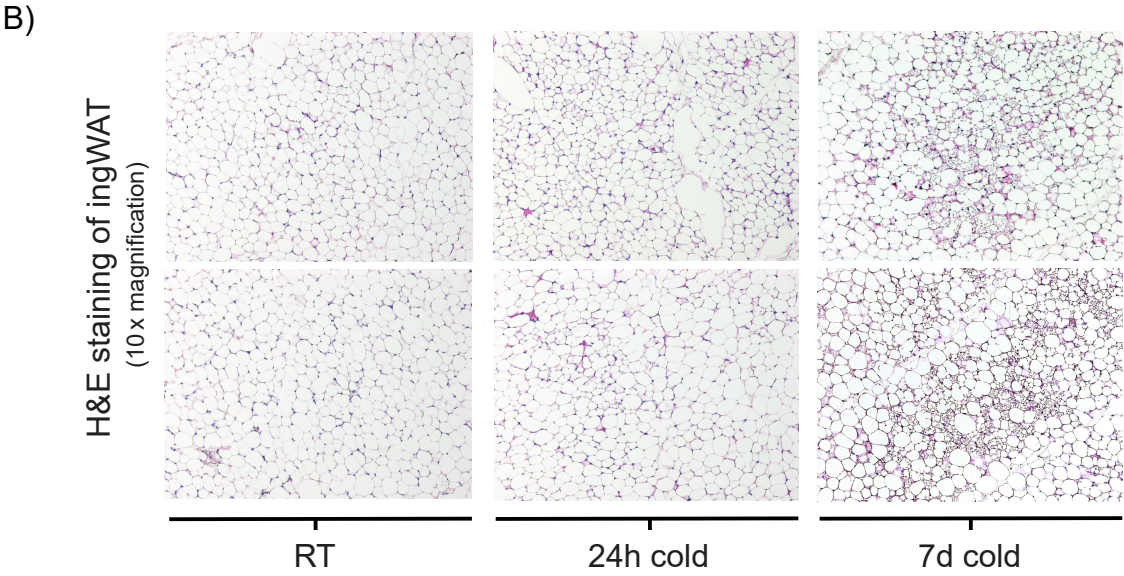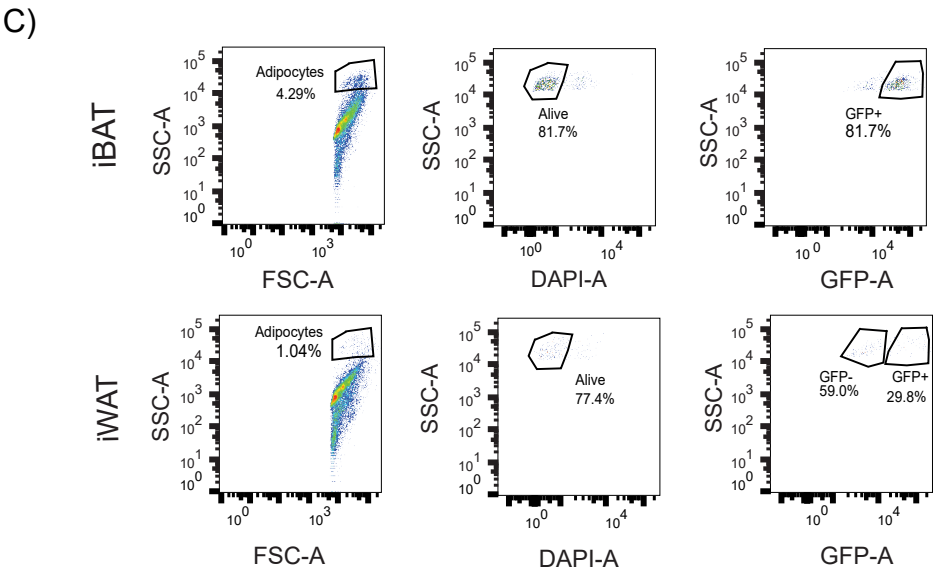

Figure S2

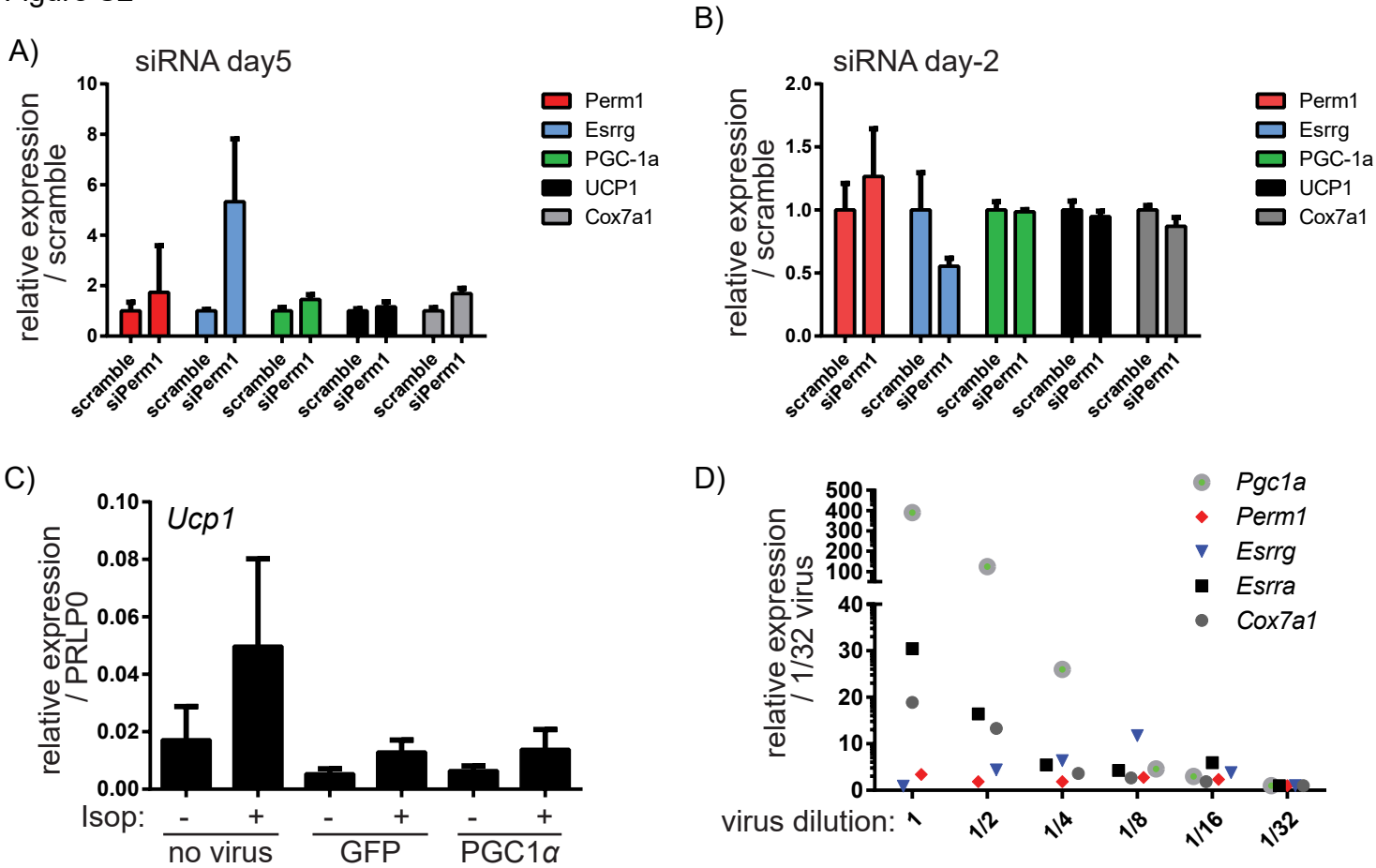

Figure S3

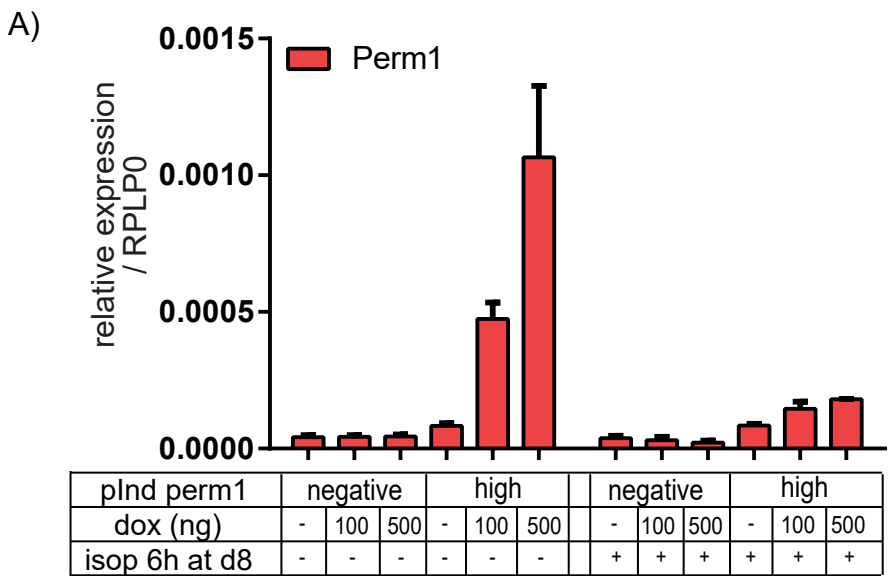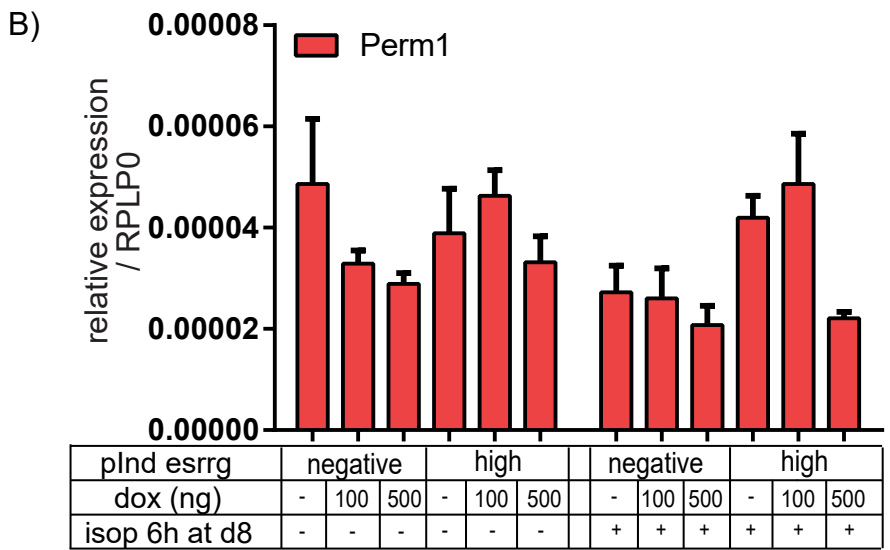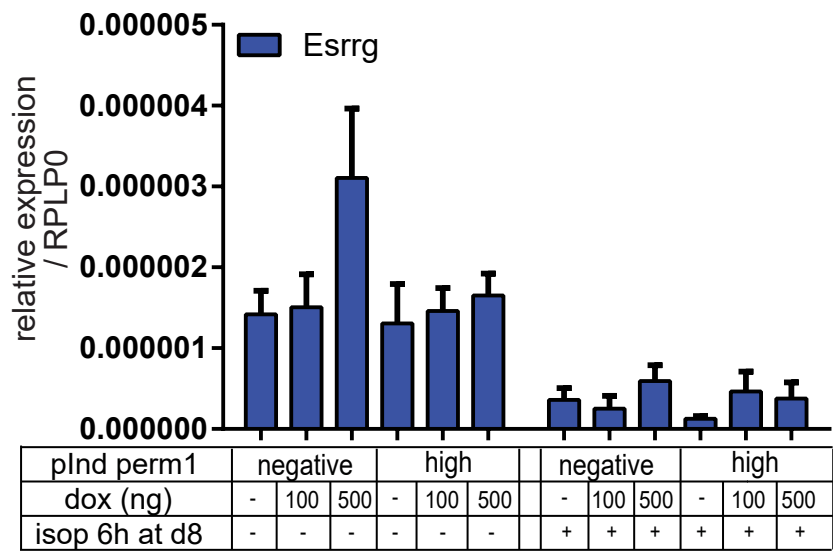

Figure S4

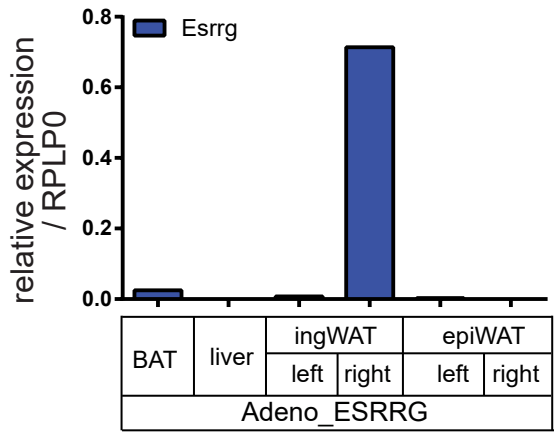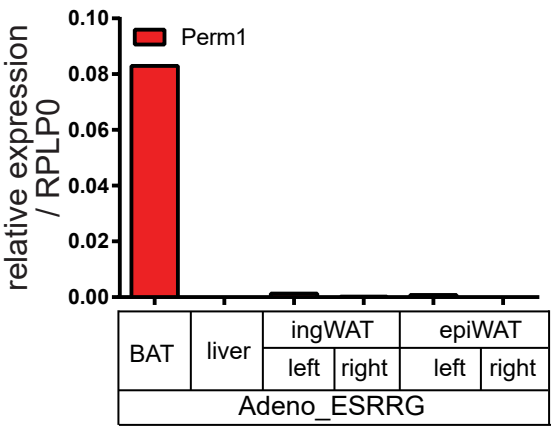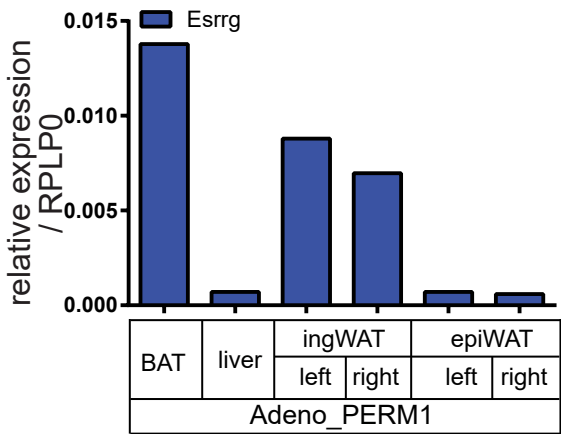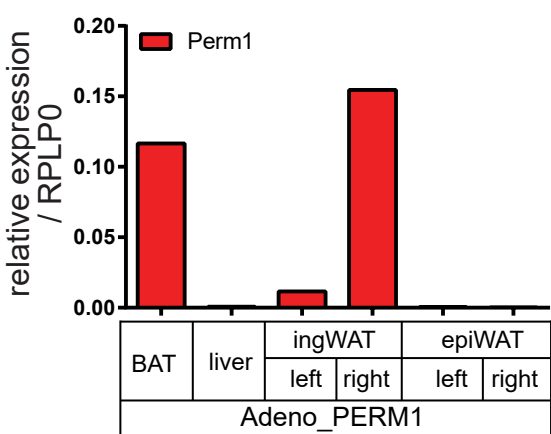

Supplement: Figure S1 — Ucp1 expression in ingWA of individual mice during cold exposure. (A) Ucp1 expression levels in the adipocyte fraction of ingWA of individual mice house at room temperature or cold exposed for 24 h or 7 days, respectively. Dotted line separates groups at room temperature with low or high Ucp1 expression. Data is presented as log2 microarray signal intensity. (B) Representative immunohistochemical images of inguinal WAT (H&E stained) from a different cohort of mice housed at room temperature (RT), 24 h or 7 days at 8°C. Major morphological changes are visible after 7 days of cold exposure, only minor changes after 24 h. Micrographs were taken at 10x magnification. (C) Representative FACS plots from interscapular BAT (iBAT) and inguinal WAT (iWAT), and gating strategy for GFP+ brown adipocytes, GFP+ brite and GFP− white adipocytes. [file Data_Sheet_1.PDF]
