# Supplementary material for: ESRRG and PERM1 Govern Mitochondrial Conversion in Brite/Beige Adipocyte Formation
Source: Front Endocrinol (Lausanne). 2020 Jun 12;11:387. doi: 10.3389/fendo.2020.00387 (PMC7304443; doi:10.3389/fendo.2020.00387)
Supplement: Supplementary file 2 [file Table_1.pdf]

Supplemental Table 1: Sequence of qPCR primers used in the study.

**Primers**	
Ucp1	FWD: GGGCATTCAGAGGCAAATCAGCTT<br>REV: ACACTGCCACACCTCCAGTCATTA
Cidea	FWD: ACTTCCTCGGCTGTCTCAATGTCA<br>REV: TCAGCAGATTCCTTAACACGGCCT
Perm1	FWD: GCTTGGAAAACAGTCTTGCTC<br>REV: GATCTCGGTCTGCCACTATG
Esrrg	FWD: ACTTGGCTGACCGAGAGTTG<br>REV: GCCAGGGACAGTGTGGAGAA
Pgc1a	FWD: ACCCCAAAGGATGCGCTCTCGTT<br>REV: TGCGGTGTCTGTAGTGGCTTGATT
Cox7a1	FWD: CAGCGTCATGGTCAGTCTGT<br>REV: AGAAAACCGTGTGGCAGAGA
Pparg	FWD: CCATTCTGGCCCACCAAC<br>REV: AATGCGAGTGGTCTTCCATCA
